# Analysis of the Borehole Effect in Borehole Radar Detection

**DOI:** 10.3390/s20205812

**Published:** 2020-10-14

**Authors:** Wentian Wang, Sixin Liu, Xuzhang Shen, Wenjun Zheng

**Affiliations:** 1 School of Earth Science and Engineering, Sun Yat-Sen University, Zhuhai 519082, China; wangwt28@mail.sysu.edu.cn (W.W.); shenxzh5@mail.sysu.edu.cn (X.S.); zhengwenjun@mail.sysu.edu.cn (W.Z.); 2 College of Geo-Exploration Science and Technology, Jilin University, Changchun 130026, China

**Keywords:** directional borehole radar, influence of nonuniformity around borehole, antenna mutual coupling, FDTD, resonance

## Abstract

The directional borehole radar can accurately locate and image the geological target around the borehole, which overcomes the shortcomings that the conventional borehole radar can only detect the depth of the target and the distance from the borehole. The directional borehole radar under consideration consists of a transmitting antenna and four receiving antennas equally distributed on the ring in the borehole. The nonuniformity caused by the borehole and sonde, as well as the mutual coupling among the four receiving antennas, will have a serious impact on the received signal and then cause interference to the azimuth recognition for the targets. In this paper, Finite difference time domain (FDTD), including the subgrid, is applied to study these effects and interferences, and the influence of borehole, sonde, and mutual coupling among the receiving antennas is found. The results show that, without considering the sonde and the fluid in the borehole, the one transmitting and one receiving borehole radar system does not have resonance, but the wave pattern of the reflected wave will have obvious distortion. For the four receiving antennas of the borehole radar system, there is obvious resonance, which is caused by the multiple reflections between the receiving antennas. However, when the fluid in the borehole is water and the relative permittivity of the sonde is low to a certain extent, the resonance disappears; that is, the generation of resonance requires a large relative permittivity material between the receiving antennas. When the influence of the sonde is considered, the resonance disappears because the relative permittivity of the sonde is low, which makes the propagation speed of the electromagnetic wave between the antennas accelerate and lose the conditions for resonance. In addition, the diameters of the sonde and the circular array of the receiving antennas can affect the received signal: the higher the diameter of the sonde and the higher the diameter of the circular array are, the better the differentiation of the received signal. The development of the research provides scientific guidance for the design and application of borehole radar in the future.

## 1. Introduction

Ground penetrating radar (GPR) is an effective means of underground detection. GPR is a geophysical method that can obtain geological information by transmitting a high frequency electromagnetic wave and analyzing the reflected wave [1]. The main working frequency of GPR is between 1 MHz and 1 GHz. The dielectric constant and conductivity are the two most important parameters that affect GPR detection. The larger the difference of the dielectric constant and conductivity between the target and the surrounding rock, the more obvious the reflection is, and the easier it is to detect the reflection. GPR is widely used in archaeology, glaciology, mineral exploration, hydrogeology, and other fields because of its advantages of nondestructive fast and convenient detection. GPR plays an increasingly important role in near-surface exploration [2,3].

Borehole radar is a kind of ground-penetrating radar. Its most important feature is that it can approach the target, overcoming the disadvantage that the ground radar’s detection depth is too shallow and can only detect near the surface [4,5,6]. The frequency of borehole radar is approximately 10 MHz to 1 GHz [7]. The measurement methods of borehole radar include single hole measurement and cross-hole measurement [8]. The developmental history of borehole radar is as follows: in 1977, Cook used borehole radar to explore coal seam for the first time [9]; in 1978, Rubin, Fowler, and Marino carried out borehole radar experiments in a diabase area [10]; in 1980, they launched the international stripa program, which developed and completed the Random Access Method of Accounting and Control(RAMAC )borehole radar system in Sweden [11]. After the 1990s, Sato M. et al. from Tohoku University of Japan researched and developed a polarimetric borehole radar system, which can carry out full polarization measurement underground [12,13,14,15]; in 2001 and 2004, Mason used borehole radar to mine in South Africa and have detailed orebody mapping [16,17].

The transmitting antenna and receiving antenna of the traditional borehole radar are omnidirectional antennas, so only the depth of the target and the distance from the borehole can be determined. However, in many projects, we need to know not only the depth of the target and the distance from the borehole but also the direction of the target. In this way, we can only use the directional borehole radar system [18,19].

The directional borehole radar system is a borehole radar system that can determine the precise orientation of the target. The directional borehole radar system is divided into two main categories: one category is the use of the array antenna to determine the precise orientation of the target by analyzing the different phases of the waveforms received by each receiving antenna; the other category is the use of the directional transmission antenna to transmit the electromagnetic waves around the borehole [20,21,22,23].

Borehole, fluid in the borehole, sonde, and the mutual coupling among the antennas can cause interference to the received signal. Therefore, we need to eliminate the influence of the borehole nonuniformity. In this regard, predecessors have also done some research. In 2007, Ebihara studied the effect of the deviation of the dipole antenna from the borehole center on the azimuth estimation results [24]. In 2015, Satoshi studied the influence of the length of the borehole, sonde, and central cylinder on the azimuth recognition and designed experiments successively with the length of the central cylinder of 160 and 40 cm. In 2015, Ebihara studied the length of the borehole, sonde, and influence of the central cylinder on azimuth recognition. He designed experiments on the effects of the borehole and the probe tube on the received signals under different central cylinder lengths [25].

The directional borehole radar studied in this paper is composed of one transmitting antenna and four receiving antennas. The four antennas are distributed in a circular ring position perpendicular to the borehole, with equal angle distribution. The Finite difference time domain (FDTD) algorithm with subgrid is used to simulate the coupling among the borehole, the fluid, the sonde, and the antenna and determine the influence of the borehole nonuniformity on the received signal.

## 2. Materials and Methods

### 2.1. Materials

**Figure 1:**
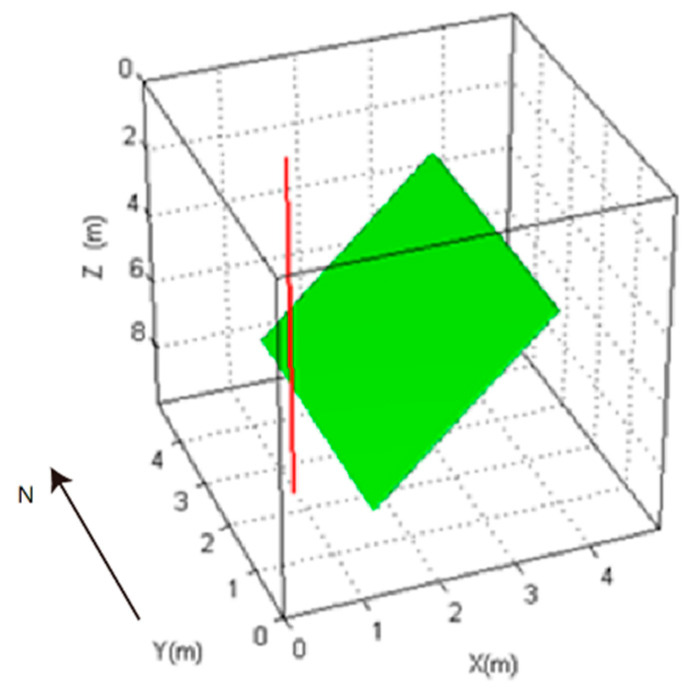
Model built to study the borehole non-uniformity influence on the received array signals (the red line represents for borehole).

**Figure 2:**
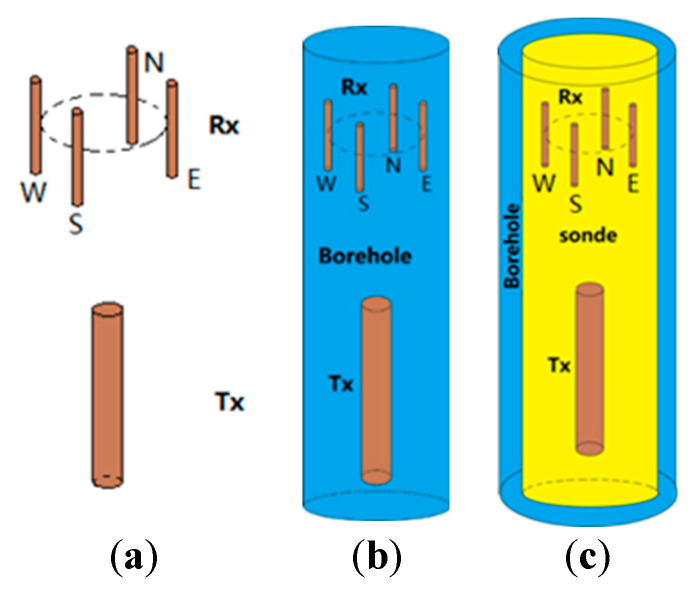
Directional borehole radar schematic diagram (both transmitting and receiving antennas are linear antennas in simulation), (**a**) schematic diagram of directional borehole radar in a uniform medium, (**b**) directional borehole radar diagram including only the borehole, and (**c**) directional borehole radar diagram including both the borehole and sonde.

The simulation model in this paper includes the influence of the borehole, the fluid in the borehole, the sonde, and the mutual coupling among the receiving antennas on the receiving signal. The linear antenna was selected as the simulation antenna in this paper. The model is shown in Figure 1. In this paper, numerical simulation is carried out for the following cases:

(1) The received signal waveforms of four receiving antennas in a uniform medium, as is shown in Figure 2a.
(2) Considering only the borehole without considering the sonde: (a) whether the borehole has an influence on the received signal; (b) the influence of the relative dielectric constant of the fluid in the borehole on the received signal; (c) the influence of the array diameter of the receiving antenna on the received signal, as is shown in Figure 2b.
(3) At the same time, in the case of the borehole and the sonde: (a) whether the sonde has an influence on the received signal; (b) if the sonde exists, the influence of the relative dielectric constant of the fluid in the borehole on the received signal; (c) the influence of the sonde diameter on the received signal, as is shown in Figure 2c.

### 2.2. Methods

**Figure 3:**
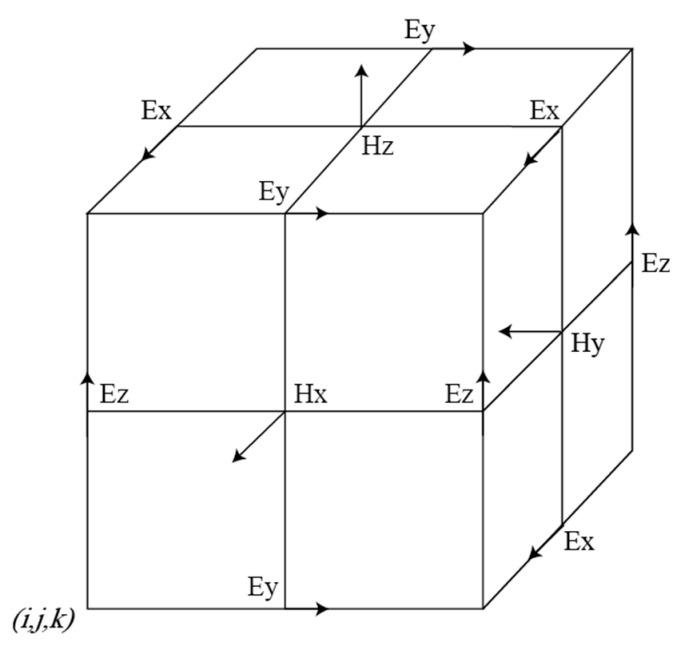
Schematic diagram of electric and magnetic field components of Yee cell in FDTD.

In this paper, we used the FDTD (finite difference time domain) method with a subgrid to simulate the directional borehole radar. The FDTD method is effective for solving Maxwell equations. FDTD obtains a numerical solution by differential treatment of the Maxwell equations. As is shown in Figure 3, the specific method is to subdivide the solution space into grids and use difference instead of partial derivative to obtain the electric and magnetic field values on each grid node, etc., and then obtain the electric and magnetic field values at each point in the space. The FDTD method has been widely used. However, there are still some flaws.

The accuracy of the traditional FDTD method depends on the size of the grid. The smaller the grid, the more accurate the FDTD method is. However, the amount of computation and the speed of computation are greatly increased. When the target is small, to a certain extent, the FDTD method needs very small grids to describe the target, which leads to a large amount of computation. In this paper, the FDTD method with a subgrid was used, that is, the subgrid was used in the area that needs accurate description, and the main grid was used in the area that does not need high-precision description. In short, in the whole region, we used the main grid to calculate once and then used the subgrid to describe once in the region that needs to be accurately depicted and finally combine the two results.

**Figure 4:**
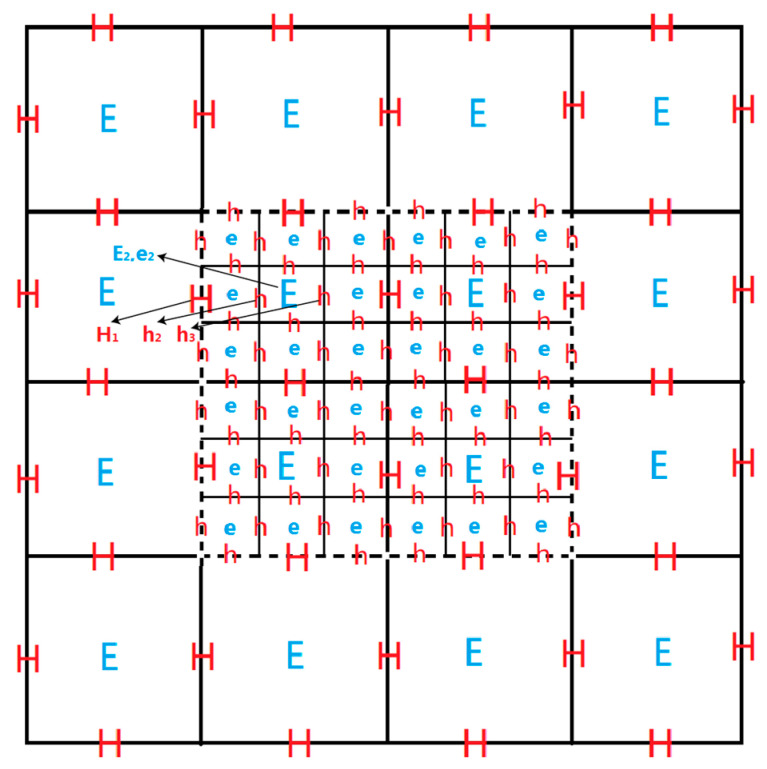
Two-dimensional slicing of main grid and subgrid.

The subgrid technology uses the subgrid only in the areas that need to be accurately described, but it uses the main grid in other areas, as is shown in Figure 4. Therefore, the subgrid technology greatly reduces the amount of calculation, greatly improves the operation speed, and saves much operation time for electromagnetic simulation. At the same time, due to the use of the subgrid in the area that needs to be accurately depicted, the required accuracy can be achieved, and the precise characterization of structure is complete [26,27].

## 3. Results

### 3.1. Numerical Model Parameters

As is shown in Figure 1,the model parameters are as follows: the number of the main grid is 200 × 200 × 200; the grid size of the main grid is 0.025 × 0.025 × 0.025 m; and the subgrid is from the 96th grid to the 105th grid in both the *X*- and *Y*-directions and from the 10th grid to the 190th grid in the Z-direction. The size ratio of main grid to the subgrid is 5:1.

The distance between the top of the fracture and the borehole is 4.25 m, and the distance between the bottom of the fracture and the borehole is 2.75 m. The depth of the top of the fracture is 1.5 m, and the depth of the bottom of the fracture is 9.25 m. The width of the fracture in the north–south direction is 1 m, and the thickness of the fracture is 0.025 m. The relative permittivity of the water in the fracture is 81, and the conductivity of the water in the fracture is 0.003 s/m. The relative permittivity of the surrounding rock is 7, and the conductivity of the surrounding rock is 0.001. The borehole diameter is 0.1 m, and the conductivity of the borehole water is 0.0072 s/m.

### 3.2. Borehole Impact

In the directional borehole radar system, the borehole will have a significant impact on the received signal. In this section, the influence of the borehole on the received signal is studied without considering the sonde. The borehole model is shown in Figure 2b. Although the situation that only considers the borehole and not considering the sonde does not exist in reality, this paper can regard it as an ideal situation to study, to pave the way for a more complex situation closer to the actual situation model.

#### 3.2.1. One Transmitting and Four Receiving Directional Antennas without a Borehole

**Figure 5:**
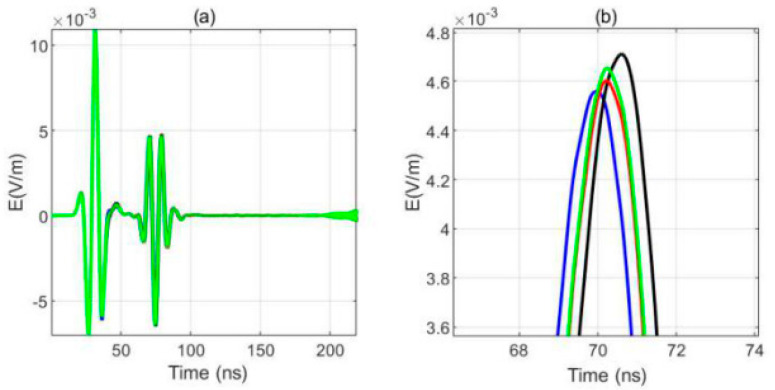
Waveform of a single-transmitting and four receiving signals without a borehole (blue: the east antenna; black: the west antenna; red: the south antenna, green: the north antenna) (**a**)Receiving waveform, (**b**) Local amplification for (**a**) (Figure 2a).

Under the condition of no borehole, the one transmitting and four receiving signals are shown in Figure 5. The figure shows that the east receiving antenna receives the signal first, the south and north receiving antennas receive the signal almost at the same time, and the west receiving antenna finally receives the signal. The received signals of the four receiving antennas can be distinguished effectively.

#### 3.2.2. The Influence of the Borehole on the One Transmitting and Four Receiving Directional Borehole Radar

**Figure 6:**
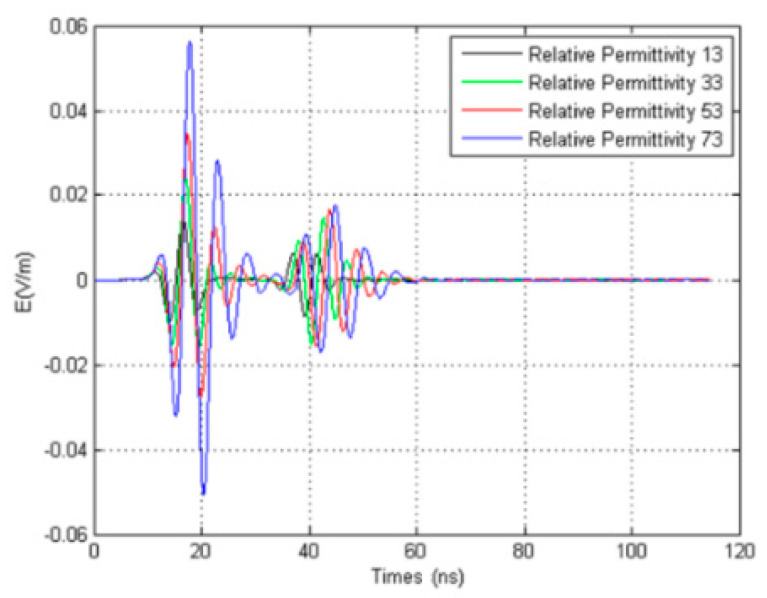
Comparisons of different dielectric constants of fluids in boreholes (Figure 2b).

In this case, the permittivity of the fluid in the borehole is 13, 33, 53, and 73 for numerical simulation. The results are shown in Figure 6. The figure shows that with the increase in the dielectric constant, the attenuation decreases, the signal of the direct wave increases significantly, and the waveform of the direct wave has some degree of distortion because the larger the dielectric constant is, the smaller the electromagnetic loss and the greater the energy of the transmitted wave. Therefore, the greater the energy of the reflected wave is, the greater the intensity of the direct wave signal. Due to the superposition of the direct wave and the reflected wave, the waveform of the direct wave is affected, so the waveform of the direct wave also changes. We can also conclude from the figure that the higher the dielectric constant, the later the reflected signal is because the slower the propagation speed of the reflected wave is, the longer the travel time for the reflected wave to reach the receiving antenna is, so the later the signal is. Therefore, we can reach the following conclusion: the larger the dielectric constant of the fluid in the borehole is, the stronger the direct wave signal, and the later the reflection signal is, the greater the distortion of the waveform caused by superposition.

#### 3.2.3. Influence of the Array Diameter of the Receiving Antenna on the Receiving Signal

**Figure 7:**
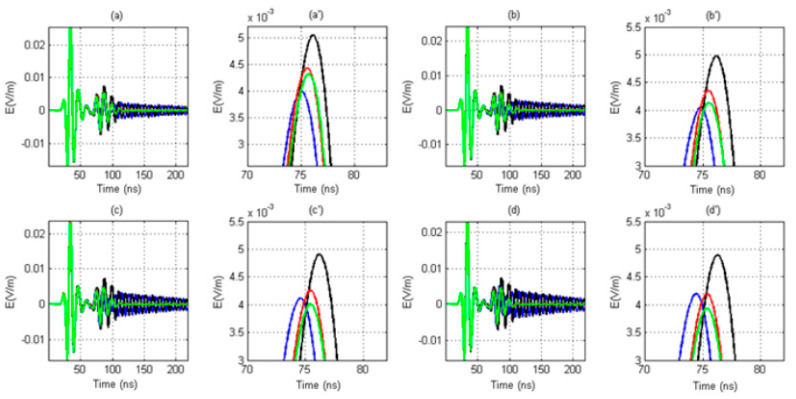
Received signals of the four receiving antennas (blue: the east antenna; black: the west antenna; red: the south antenna, green: the north antenna) for different array diameters. (**a**) 4 cm, (**b**) 5 cm, (**c**) 6 cm, (**d**) 7 cm. (**a’**–**d’**) Local amplification for corresponding figures (**a**–**d**).

In this experiment, array diameters of 4, 5, 6, and 7 cm were selected for a numerical simulation experiment. The results are shown in Figure 7a–d. From the results, for different size diameters of the receiving antenna array, the waveforms received by the east receiving antenna arrive first, the waveforms received by the west receiving antenna arrive last, and the waveforms received by the south receiving antenna and the north receiving antenna arrive at the same time. Therefore, the received signals have good discrimination. However, the influence of the array diameter of the receiving antenna on the discrimination of the received waveforms of the four antennas is still obvious. In the presence of a borehole, the sequence of the waveforms received by the array antennas of various diameters is interfered with to some extent. The larger the array antenna diameter is, the more obvious the signal discrimination is. At the same time, no matter what the diameter of the array antenna is, the received signals of the four receiving antennas all appear to show different degrees of distortion, and the received signals in the east–west direction appear resonant. Due to the resonance, the following section presents a detailed study. Therefore, we reached the following conclusion: the larger the array diameter of the receiving antenna is, the more distinct the four received signals are.

#### 3.2.4. The Influence of the Dielectric Constant of the Fluid in the Borehole on the Received Signal of the Array Antenna

**Figure 8:**
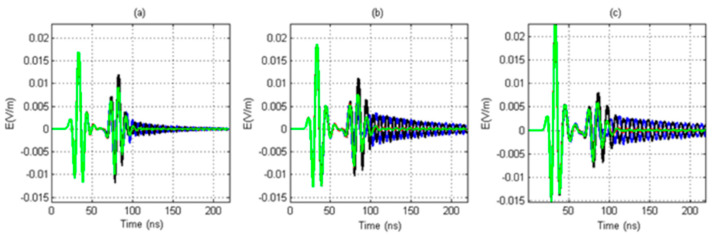
Received signal for the cases of in-borehole dielectric constants (**a**) 33, (**b**) 53, and (**c**) 73.

In this simulation, the influence of the dielectric constant of the fluid in the borehole on the received signal of the array antenna was studied without considering the sonde. In this study, the permittivity of the fluid in the borehole is 33, 53, and 73, as is shown in Figure 8. The array diameter of the receiving antenna is 5 cm. The simulation results are shown in Figure 8a–c. The figure shows that the greater the dielectric constant of the fluid in the borehole, the more obvious the resonance of the signal received by the east–west receiving antenna is, while the signal received by the north–south receiving antennas does not appear to show an obvious resonance phenomenon. At the same time, there is no resonance in the previous one transmitting and four receiving directional borehole radar system because the resonance is caused by the back and forth oscillation of the reflected wave between the receiving antennas. As there is only one receiving antenna in the single transmitting and single receiving borehole radar system, there will not be back and forth oscillation between the receiving antennas, and the resonance disappears. From Figure 6, the larger the dielectric constant of the fluid in the borehole, the more obvious the resonance is, because when the dielectric constant is larger, the propagation speed of the electromagnetic wave will be slower, and then the resonance will be generated between the receiving antennas.

#### 3.2.5. Analysis of the Cause of the Resonance of the Receiving Signal

**Figure 9:**
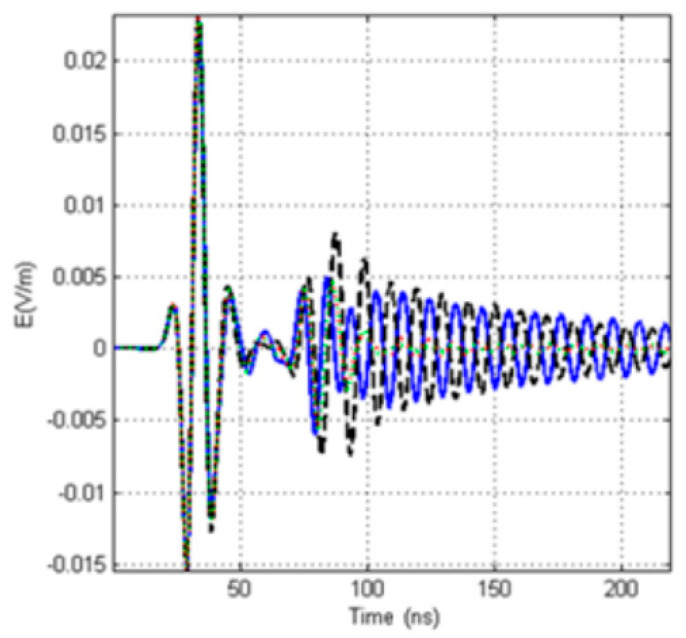
The received signal when the dielectric constant of the borehole fluid is 81. (blue: the east antenna; black: the west antenna; red: the south antenna, green: the north antenna).

**Figure 10:**
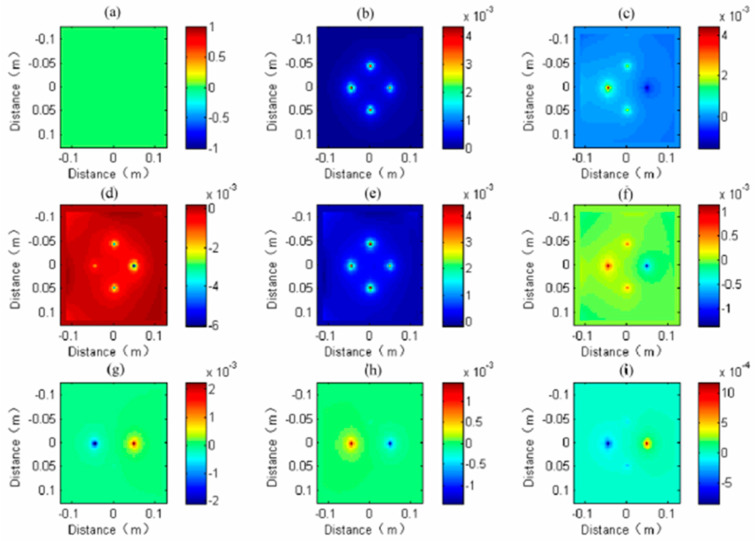
Wave field snapshots in subgrid region, (**a**) 17 ns, (**b**) 34 ns, (**c**) 66 ns, (**d**) 76 ns, (**e**) 86 ns, (**f**) 96 ns, (**g**) 116 ns, (**h**) 136 ns, (**i**) 156 ns.

The Figure 8 shows that resonance will occur when considering the borehole and the single transmission and four receiving antennas of the linear antenna. The cause of the resonance was analyzed. The array diameter of the receiving antenna is 9 cm, and the relative permittivity of the fluid in the borehole is 81. The result is shown in Figure 9. To intuitively show the cause of the resonance, we selected a snapshot of the plane where the receiving antenna center is located in the subgrid area for display, as shown in Figure 10.

From the wave field snapshot of 17 ns, as is shown in Figure 10a, we can see that the direct wave does not arrive at this time; from the wave field snapshot of 34 ns, as is shown in Figure 10b, we can see that four anomalies are obvious: the location of the receiving antenna and the centers of the four anomalies are maxima with roughly the same intensity. As Figure 10b shows, the direct wave has arrived, and four receiving antennas receive the signals at the same time. In Figure 10c, the reflected wave has arrived because there is a peak in the location of the west receiving antenna. In Figure 10d–f, the reflected wave propagates back and forth in the east–west receiving antennas. In Figure 10g–i, because of the attenuation of the reflected wave, we can see peak only in the east–west receiving antennas because the target is in the east direction of the borehole.

Therefore, we can conclude that the resonance between the east and west receiving antennas is caused by the back and forth oscillation of the reflected wave between the east and west receiving antennas.

### 3.3. Sonde Impact

In production, the directional borehole radar system is located in the sonde, which has a relatively low dielectric constant. Therefore, the inhomogeneity of the dielectric constant is caused by the existence of the sonde. This kind of nonuniformity will still affect the received signals of four receiving antennas. This section reports a detailed study on the influence of the sonde. The borehole model simulated in this section is shown in Figure 2c.

#### 3.3.1. Receiving a Signal in a Uniform Medium

**Figure 11:**
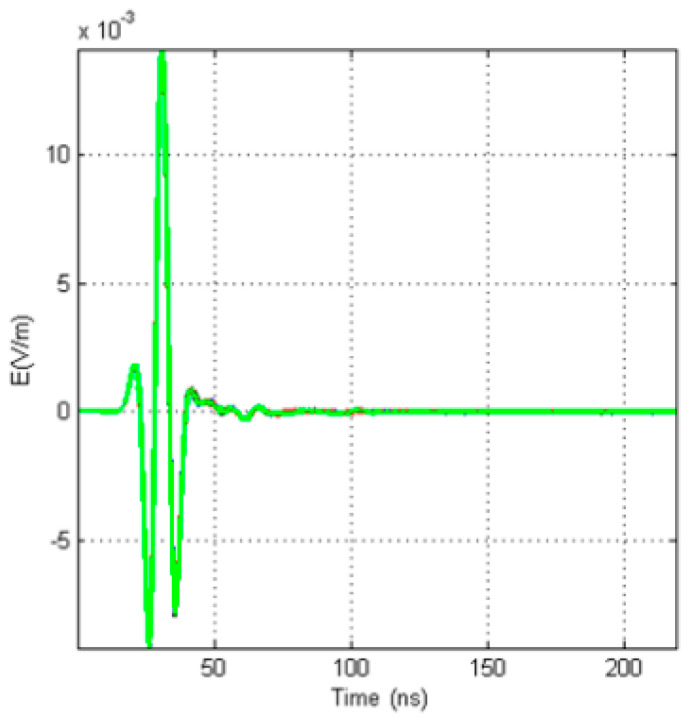
Received signals of four receiving antennas in homogeneous media.

In this simulation, the relative permittivity of the fluid in the borehole is 81, and the permittivity of the sonde is 3. In this case, the signals received by the four receiving antennas are as shown in Figure 11. The signals received by the four receiving antennas arrive at the same time without resonance, and only one direct wave is observed.

#### 3.3.2. The Influence of the Sonde Diameter on the Receiving Signal

**Figure 12:**
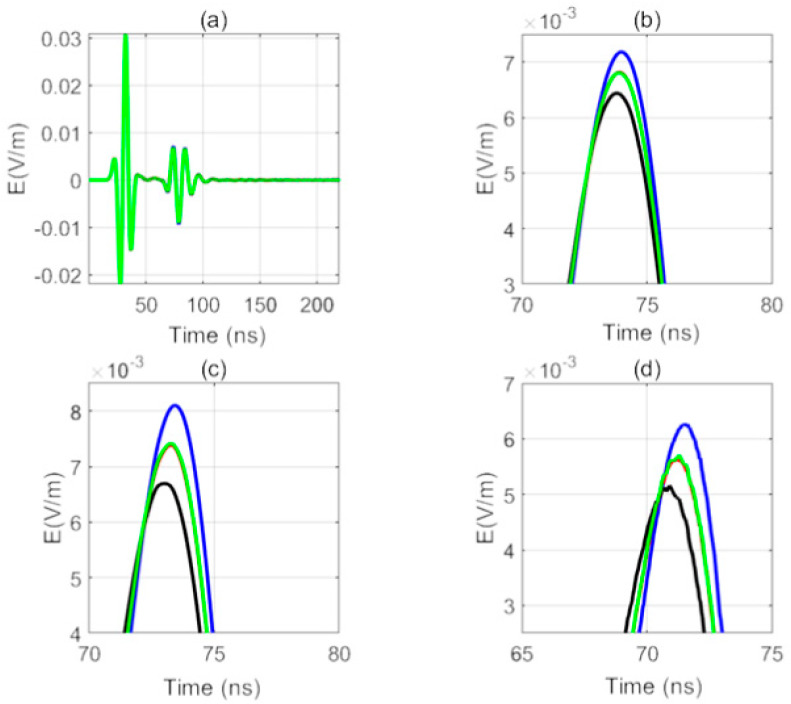
Received signals for sondes with different diameters (blue: the east antenna; black: the west antenna; red: the south antenna, green: the north antenna). (**a**) Received signals for a sonde diameter of 3 cm, (**b**) local amplification for (**a**), (**c**) local amplification for a sonde diameter of 5 cm, and (**d**) local amplification for a sonde diameter of 7 cm.

The size difference of the sonde diameter will also have a serious impact on the received signal. In this simulation, the relative permittivity of the sonde is 3, and the conductivity is 0.000001 s/m. In the process of production, the relative permittivity of the borehole water is usually 81, so the relative permittivity of borehole water selected in this simulation is 81. In this simulation, the borehole diameter is 10 cm and the sonde diameter is 3, 5, and 7 cm. The simulation results are shown in Figure 12a–d.

When the sonde exists, the resonance in the received signal disappears because the relative permittivity of the sonde is small, which makes the propagation speed of the electromagnetic wave between the antennas faster and loses the condition of resonance generation. For sondes of different diameters, the first wave is received by the east receiving antenna, the last wave is received by the west receiving antenna, and the wave is received by the south receiving antenna and the north receiving antenna at the same time. As the fracture is in the east direction, the received signal is consistent with the model. In Figure 12b, the peak sequence of the received signals of the four receiving antennas is not obvious. In Figure 12d, the order of the peaks of the received signals of the antenna is clearly distinguished. Therefore, the larger the sonde diameter is, the more distinct the discrimination is.

We can conclude that the existence of the sonde can eliminate the influence of resonance between antennas, and the larger the diameter of sonde is, the better the four receiving antennas can be distinguished.

#### 3.3.3. The Influence of the Dielectric Constant of the Fluid in the Borehole on the Signal Received by the Antenna in the Sonde

**Figure 13:**
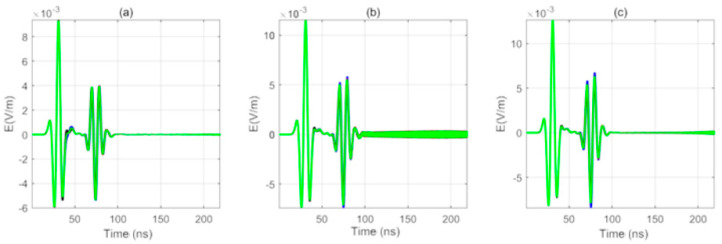
Received signals for borehole fluid with different dielectric constants (blue: the east antenna; black: the west antenna; red: the south antenna, green: the north antenna); (**a**) the dielectric constant is 11, (**b**) the dielectric constant is 41, and (**c**) the dielectric constant is 71.

When considering the borehole and the sonde, the different dielectric constants of the fluid in the well will have a certain impact on the received signal. This simulation studies the influence of the dielectric constant of the fluid in the borehole on the signal received by the antenna in the sonde. In this simulation, when the dielectric constant of the fluid in the borehole is 11, 41, and 71, the diameter of the sonde is 7 cm, the relative dielectric constant of the sonde is 3, and the array diameter of the receiving antenna is 6 cm. The simulation results are shown in Figure 13a–c. The larger the dielectric constant of the fluid in the borehole is, the stronger the signal of the direct wave and the reflected wave. Therefore, the greater the dielectric constant of the fluid in the borehole is, the greater the intensity of the direct wave and the reflected signal.

#### 3.3.4. The Influence of the Dielectric Constant of the Sonde on the Received Signal

**Figure 14:**
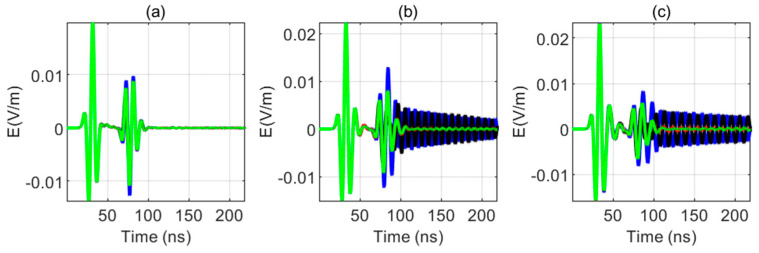
Received signals of the four receiving antennas for sondes with different dielectric constants (blue: the east antenna; black: the west antenna; red: the south antenna, green: the north antenna); (**a**) the sonde dielectric constant is 13, (**b**) the sonde dielectric constant is 43, and (**c**) the sonde dielectric constant is 73.

The difference in the dielectric constant of the sonde will change the propagation speed of the electromagnetic wave between the receiving antennas, so it will have a great impact on the received signal. In this simulation study, the influence of different permittivity sondes on the received signal is considered. The borehole water is selected as the fluid in the borehole, i.e., the relative permittivity is 81. The diameter of the sonde is 7 cm, and the diameter of the receiving antenna array is 6 cm. In this simulation, the relative permittivity of the sonde is 13, 43, and 73. Although the permittivity of the sonde is rarely higher than 43 in production, as an ideal condition to find the influence of the probe permittivity on the received signal, we still need to simulate. The simulation results are shown in Figure 14a–c.

From Figure 14a–c, we can see that as the dielectric constant of the sonde increases, the resonance between the east–west receiving antennas increases because the increase in the dielectric constant of the sonde leads to the decrease in the propagation speed of the electromagnetic wave between the antennas, and the reflection wave oscillates back and forth between the east–west receiving antennas. The resonance seriously interferes with the reflection information. Therefore, the larger the dielectric constant of the sonde is, the stronger the resonance between the antennas is.

## 4. Discussion

There are many problems in the study of the nonuniformity around the directional borehole radar. In this paper, the influence of the borehole, fluid, sonde, array diameter, and mutual coupling between receiving antennas was studied.

From the simulation results without considering the sonde, the influence of borehole on the borehole radar can be determined. The higher the dielectric constant, the later the reflected signal is because the higher the dielectric constant, the slower the propagation speed of the reflected wave, and the longer the travel time for the reflected wave to reach the receiving antenna, so the later the signal is. We can reach the following conclusion: the larger the dielectric constant of the fluid in the borehole is, the stronger the direct wave signal, and the later the reflection signal is, the greater the degree of distortion of the waveform caused by superposition.

For different sizes of receiving antenna array diameter, the waveforms received by the east receiving antenna arrive first, the waveforms received by the west receiving antenna arrive last, and the waveforms received by the south receiving antenna and the north receiving antenna arrive at the same time. Therefore, the received signals have good discrimination. However, the influence of the array diameter of the receiving antenna on the discrimination of the received waveforms of the four antennas is still obvious. In the presence of a borehole, the sequence of the waveforms received by the array antennas of various diameters is interfered with to some extent. The larger the array antenna diameter is, the more obvious the signal discrimination is. At the same time, no matter what the diameter of the array antenna is, the received signals of the four receiving antennas all appear to show different degrees of distortion, and the received signals in the east–west direction appear resonant. The snapshot shows that the generation of resonance has obvious directionality; that is, the reflected wave is formed by repeated resonance between two antennas in the direction of the incoming wave.

The larger the dielectric constant of the fluid in the borehole is, the more obvious the resonance of the signal received by the east–west receiving antenna is, while the resonance of the signal received by the north–south receiving antennas does not appear. The reason is that the larger the dielectric constant is, the slower the electromagnetic wave propagation is, so the electromagnetic wave is more likely to form vibration between the receiving antennas. At the same time, because the target is in the east–west direction, the reflected signal is more likely to oscillate between the receiving antennas in the east–west direction. Therefore, we conclude that the larger the dielectric constant of the fluid in the borehole is, the more obvious the resonance is.

When the sonde exists, the resonance in the received signal disappears because the relative permittivity of the sonde is small, which makes the propagation speed of the electromagnetic wave between the antennas faster and loses the condition of resonance generation. For sondes of different diameters, the first wave is received by the east receiving antenna, the last wave is received by the west receiving antenna, and the wave is received by the south receiving antenna and the north receiving antenna at the same time. Therefore, the larger the sonde diameter is, the more distinct the discrimination is. The existence of the sonde can eliminate the influence of resonance between antennas, and the larger the sonde diameter is, the better the four receiving antennas can be distinguished.

When considering the borehole and the sonde together, the different dielectric constants of the fluid in the borehole will have a certain impact on the received signal. The larger the dielectric constant of the fluid in the borehole is, the stronger the signal of the direct wave and the reflected wave is. Therefore, the greater the dielectric constant of the fluid in the borehole is, the greater the intensity of the direct wave and the reflected signal is.

With the increase in the dielectric constant of the sonde, the resonance between the east–west receiving antennas increases because the increase in the dielectric constant of the probe leads to the decrease in the propagation speed of the electromagnetic wave between the antennas, and the reflection wave oscillates back and forth between the east–west receiving antennas. The resonance seriously interferes with the reflection information. Therefore, the larger the dielectric constant of the probe is, the stronger the resonance between the antennas is.

## 5. Conclusions

In this paper, the FDTD method with subgrid was used to simulate the mutual coupling among the borehole, the sonde, and the receiving antenna.

First, this paper studied the effect of the borehole on the received signal. When there is only one transmitting antenna and one receiving antenna in the borehole, the dielectric constant of the fluid in the borehole has a significant effect on the received signal. The larger the dielectric constant of the fluid in the borehole is, the stronger the direct wave signal, the later the reflection signal, and the more serious the waveform distortion. When there is one transmitting antenna and four receiving antennas in the borehole, the array diameter of the receiving antenna and the dielectric constant of the fluid in the borehole have an obvious influence on the received signal. The larger the receiving antenna array diameter is, the better the discrimination of the received signal is. The larger the relative permittivity of the fluid in the borehole is, the stronger the resonance in the received signal is. Resonance is formed by the reflected wave oscillating back and forth between the receiving antennas.

Then, this paper studied the influence of the sonde on the received signal considering the borehole condition. The larger the sonde diameter is, the better the discrimination of the received signal is. The larger the relative permittivity of the fluid in the borehole is, the stronger the direct wave and the reflected signal is. The larger the relative permittivity of the sonde is, the more obvious the resonance phenomenon in the received signal is.

## References

[1] Bristow C.S., Jol H.M. (2003). Ground Penetrating Radar in Sediments.

[2] Daniels D.J. (2004). Ground Penetrating Radar.

[3] Jol H.M. (2009). Ground Penetrating Radar Theory and Applications.

[4] Olsson O., Falk L., Forslund O., Lundmark L., Sandberg E. (1992). Borehole radar applied to the characterization of hydraulically conductive fracture zones in crystalline rock. Geophys. Prospect..

[5] Jia Z., Liu S., Zhang L., Hu B., Zhang J. (2019). Weak Signal Extraction from Lunar Penetrating Radar Channel 1 Data Based on Local Correlation. Electronics.

[6] Liu S., Takahashi M.S.K. (2004). Application of borehole radar for subsurface physical measurement. J. Geophys. Eng..

[7] Liu S., Sato M. (2020). Electromagnetic Logging Technique Based on Borehole Radar. IEEE Trans. Geosci. Remote Sens..

[8] Sato M., Thierbach R. (1991). Analysis of a borehole radar in cross-hole mode. IEEE Trans. Geosci. Remote Sens..

[9] Cook J.C. (1977). Borehole-radar exploration in a coal seam. Geophysics.

[10] Binley A., Cassiani G., Middleton R. (2001). Vadose zone flow model parametrisation using cross-borehole radar and resistivity imaging. J. Hydrol..

[11] Falk L.R., Olsson O.L., Sandberg E.V. (1991). Combined interpretation of fracture zones in crystalline rock using single-hole, crosshole tomography and directional borehole-radar data. Log Anal..

[12] Sato M., Ohkubo T., Niitsuma H. (1995). Cross-polarization borehole radar measurements with a slot antenna. J. Appl. Geophys..

[13] Miwa T., Sato M., Niitsuma H. (1999). Subsurface fracture measurement with polarimetric borehole radar. IEEE Trans. Geosci. Remote Sens..

[14] Ebihara S., Sato M., Niitsuma H. (2000). Super-resolution of coherent targets by a directional borehole radar. IEEE Trans. Geosci. Remote Sens..

[15] Miwa T., Sato M., Niitsuma H. (2000). Enhancement of reflected waves in single-hole polarimetric borehole radar measurement. IEEE Trans. Antennas Propag..

[16] Mason I., Hargreaves J., Turner G., Wellington A. (2001). Detailed orebody mapping using borehole radar. Explor. Geophys..

[17] Mason I., Cloete J. (2004). Vhf Band Slimline Borehole Radar Experiences in The South African Mining Industry. Ground Penetrating Radar.

[18] Liu S., Sato M. (2005). Transient Radiation from an Unloaded, finite Dipole Antenna in a Borehole: Experimental and Numerical Results. Geophysics.

[19] Borchert O., Behaimanot K., Glasmachers A. (2009). Directional borehole radar calibration. J. Appl. Geophys..

[20] Sato M., Tanimoto T. A Shielded Loop Array Antenna for a Directional Borehole Radar. Proceedings of the 4th International Conference on Ground Penetrating Radar.

[21] Liu S., Sato M. (2006). Subsurface Water-filled Fracture Detection by Borehole Radar: A Case History. J. Environ. Eng. Geophys..

[22] Liu S., Wang W., Fu L., Lu Q. (2019). Linear Prediction-Based DOA Estimation for Directional Borehole Radar 3-D Imaging. IEEE Trans. Geosci. Remote Sens..

[23] Liu S., Jia Z., Zhu Y., Zhao X., Cheng S. (2019). Optimized Refraction Travel Time Tomography. Appl. Sci..

[24] Ebihara S., Hashimoto Y. (2007). MoM analysis of dipole antennas in crosshole borehole radar and field experiments. IEEE Trans. Geosci. Remote Sens..

[25] Ebihara S., Kimura Y., Shimomura T., Uchimura R., Choshi H. (2015). Coaxial-fed circular dipole array antenna with ferrite loading for thin directional borehole radar sonde. IEEE Trans. Geosci. Remote Sens..

[26] Zivanovic S.S., Yee K.S. (1991). A subgridding method for the time-domain finite-difference method to solve Maxwell’s equations. IEEE Trans. Microwave Theor. Tech..

[27] Chevalier M.W., Luebbers J.R., Cable P.V. (1997). FDTD local grid with material traverse. IEEE Trans. Antennas Propag..

